# Some Moral Aspects of the C. D. Acts

**Published:** 1897-03-06

**Authors:** 


### The Hospital

A Journal of

The Medical Sciences and Hospital Administration.

Vol. XXI.—No. 545.] [March 6, 1897.

### Some Moral Aspects of the C. D. Acts.

It seems a little trite to say that the English are a moral people; and that they will not, in the mass, move in an immoral direction if they can help it. To say that this is a good thing, a worthy and an honourable thing, is trite also. Civilisation is founded on morality; and the man who—statesman, physician, or whatever else he may be—derides, or attempts to lessen the value of national morality, is *ipso facto* demonstrated a fool.

But the study of morals, more especially in their practical application to life, and most of all in their national application, is by no means so easy as a sum in simple addition. Unfortunately there are a very great many among us who think it is. But those who have devoted time and labour to the study, the professors and students of moral philosophy, and the private individuals who are endowed with fine consciences and a keen insight into moral subtleties, know better.

The question of syphilitic contagion and its prevention, including, as it now does for us, the history of the Contagious Diseases Acts, is one of the most difficult, as it is undoubtedly one of the most important, of all the national questions of the day. If we may trust the facts recently published by Major-General Dashwood and others, and at any rate their relative correctness may certainly be trusted, there is a condition of things in our Indian army which, whatever else it does not do, must seriously give us pause. To put the main fact at a low figure, more than half the soldiers of that army suffer from syphilis. Now, what that really means the general public does not understand in the least; a majority of medical practitioners perhaps do not fully appreciate; and even those practitioners who, by virtue of a special personal experience, get the truth in some of its most miserable and fearful aspects worked into their minds—even they, we say, do not in any sense adequately lay hold of the significance of the case unless to their medical competency and experience they add a realising imagination and a philosophic mind. Syphilis is not a very painful disease; it does not immediately prostrate a person as many other diseases do. But if its deadly effect upon the constitution, both in its earlier and later stages, be fully taken into account, there are few specialists who will not endorse the contention that it is one of the most profoundly and permanently ruinous to the vital powers of all the diseases which medical science is acquainted with. In a word, syphilis in a large proportion of cases so takes the life out of a man that he is no longer fit to be a soldier; and not only so, but that which disqualifies him for soldiering makes him also so inferior in physique and capacity to the average civilian of his own class that he is no more competent, after his return to civil employment, to fight the battle of life successfully than he was able, whilst still in the army, to fight the battles of his country.

What is to be done? This is a question which affects, in the first place, the fighting capacity of our army; and, in the second, the health and life of thousands of not unmeritorious individuals. The most practical solution which strikes the writer is not, perhaps, a solution at all, but rather a way to a solution. The question is, truly and properly, one for doctors; and if it were left to them it would be solved without the delay of an hour, to the unspeakable advantage of the army and the country. Unfortunately it has been made a moral as well as, and almost more than, a physiological question. But, reverting for a moment to the innate difficulty of many moral problems, more especially those which have to be dealt with on a national scale, are we not entitled to say that the very worst and most inadequate of all the ways of settling a moral question of this order is to do it by an appeal to popular prejudice and feeling? What we suggest is a commission of experts appointed *ad hoc*; the commission to consist of physiologists, physicians, clergymen, philosophic moralists, men of business, and practical statesmen. That the subject is of sufficient importance to warrant the appointment of such a commission no one who at all realises its true significance can for a moment doubt; that the vast mass of the thoughtful public would be glad to see it removed from the arena of popular prejudice and passion it is quite certain. The question which remains to be asked is this: Would the sense and responsibility of the country really be content with the findings and advice of such a commission? We think they would; and if evidence in support of this view be asked for, we point to the undoubted calm which has settled down upon the public mind with regard to vaccination and anti-vaccination ever since the recent Commission pronounced so favourably upon the prophylactic value of Jenner's great discovery. That things should remain in their present condition is, or ought to be, impossible. As a practical people we are bound, for very shame, to make some effort to deal with them in a more rational and humane way. A commission of experts, properly selected, would let in the light of day, and purify the physical and moral atmospheres, as they have not been purified since the first day when the question began to engage public attention.

